# Case report: Personalized adapted motor activity in a COVID-19 patient complicated by critical illness polyneuropathy and myopathy

**DOI:** 10.3389/fphys.2022.1035255

**Published:** 2022-11-17

**Authors:** Oscar Crisafulli, Marta Baroscelli, Luca Grattarola, Giuseppe Tansini, Cristian Zampella, Giuseppe D’Antona

**Affiliations:** ^1^ CRIAMS-Sport Medicine Centre Voghera, University of Pavia, Voghera, Italy; ^2^ Oncology Unit Casalpusterlengo, Ospedale Maggiore di, Lodi, Italy; ^3^ Department of Public Health, Experimental and Forensic Medicine, University of Pavia, Pavia, Italy

**Keywords:** AMA, post-COVID-19, CIM, CIP, case report

## Abstract

**Background:** COVID-19 may require hospitalization in an intensive care unit (ICU) and is often associated with the onset of critical illness polyneuropathy (CIP) and critical illness myopathy (CIM). Due to the spread of the disease around the world, the identification of new rehabilitation strategies for patients facing this sequence of events is of increasing importance.

**Case presentation:** We report the clinical presentation and the beneficial effects of a prolonged, supervised adapted motor activity (AMA) program in a highly deconditioned 61-year-old male COVID-19 patient discharged from the ICU and complicated by residual CIP and CIM. The program included aerobic, strength, gait, and balance training (1 h, 2 sessions per week).

**Measures:** Pulmonary (spirometry), metabolic (indirect calorimetry and bioimpedance), and neuromuscular functions (electromyography) were evaluated at baseline and after 1 year of training.

**Results:** Relative to baseline, an amelioration of several spirometric parameters such as vital capacity (VC, +40%), total lung capacity (TLC, +25%), and forced expiratory volume in 1 s (FEV1, +28%) was appreciable. Metabolic parameters such as body water (60%–46%), phase angle (3.6°–5.9°), and respiratory quotient (0.92–0.8) returned to the physiological range. Electromyographic parameters were substantially unchanged. The overall amelioration in clinical parameters resulted in a significant improvement of patient autonomy and the quality of life.

**Conclusion:** Our results highlight the importance of AMA for counteracting respiratory, metabolic, and functional but not neuromuscular impairments in COVID-19 patients with residual CIM and CIP.

## Introduction

The search for effective rehabilitation strategies, following discharge of COVID-19 patients from an intensive care unit (ICU), is of major importance, particularly in the presence of additional complications, such as critical illness polyneuropathy (CIP) and critical illness myopathy (CIM), leading to muscle weakness and failure to wean from the ventilator (Latronico & Bolton, 2011). In a systematic review with meta-analysis (Chang et al., 2021), it was found that in a total of 28 studies comprising 12,437 COVID-19 ICU admissions, 69% of cases needed invasive mechanical ventilation (IMV). In addition, approximately 50% of critically ill patients receiving IMV for more than 7 days develop CIP and/or CIM (Z’Graggen & Tankisi, 2020). Latronico & Bolton (2011) reported that CIP and CIM increase hospital mortality in patients who are critically ill and cause chronic disability in survivors due to structural changes like axonal nerve degeneration, skeletal muscle necrosis, and myosin loss. Hence, these type of patients will not only face the long-term effects of severe COVID-19 (Torres-Castro et al., 2021) but also the symptoms of deconditioning due to CIP and CIM, making the rehabilitation process even more complex (Bagnato et al., 2021).

We present a supervised adapted motor activity (AMA) program in a case of severe COVID-19 requiring hospitalization in the ICU for 47 days, of which 20 days were with mechanical ventilation, complicated by the appearance of CIP and CIM. Before and after AMA, the patient underwent a battery of respiratory, metabolic, and electromyographic tests.

## Case description

The patient was a 61-year-old male without a relevant past medical history. At the end of February 2020, he began to show signs of acute rhinitis and very high fever. On March 3, he was admitted to the infectious diseases ward for acute COVID-19 pneumonia, and on March 5, his condition worsened, so a continuous positive airway pressure (CPAP) helmet was used. On March 7, he was transferred to the ICU and subjected to orotracheal intubation and mechanical ventilation. In the ICU, pronation cycles were performed with a gradual improvement and reduction of sedation until the recovery of consciousness. In this phase, the patient was hypotonic, a clinical sign which, together with asthenia, led doctors to hypothesize the diagnosis of CIP and CIM.

On April 22, he was transferred to the COVID pneumo-rehabilitation ward to continue treatment and respiratory weaning. On May 18, he was transferred to the neurorehabilitation ward in a condition of tetraparesis and marked weakness. Upon arrival in the neurorehabilitation ward, the patient presented preserved voluntary motility in the four limbs with severe weakness and hypotrophy, which is more marked in the lower limbs. No obvious issues were noted for the upper limbs, except for the bicep reflex which was bilaterally not elicitable and difficulty in holding in the Mingazzini test. The lower limbs were hypotrophic, with segmental asthenia of the hip flexors, bilaterally absent ankle flexion, and barely evident ankle extension. Furthermore, the Mingazzini II test, which requires the patient to lie on his back with eyes closed, with the hip and knees flexed to about 90° and holding the position for 30 s, was not executable. The trunk was hypotonic with difficulty in reaching the semi-sitting position in the bed, even with arm support.

## Diagnostic assessment

Electromyography (EMG) and electroneurography (ENG) identified the underlying myopathy and severe sensory-motor axonal neuropathy, prevalent in the lower limbs, with significant bilateral impairment of the internal (IPSN, stimulation 7 cm above the popliteal fossa crease at the midpoint between the tendons of biceps femoris (laterally) and tendons of semitendinosus and semimembranosus (medially); registration from the abductor hallucis belly below the navicular bony prominence) and external popliteal sciatic nerves (EPSN, stimulation at the tibiofibular joint; registration from the extensor digitorum communis below the external malleolus). Additionally, videofluoroscopy showed reduced swallowing function; therefore, after abdominal ultrasound and gastroenterological evaluation, on June 3, percutaneous endoscopic gastrostomy (PEG) was performed. In addition to global recovery, the rehabilitation process was aimed toward the recovery of the walking ability, oral feeding re-education until total weaning from the PEG (which was removed on August 6), and the improvement of the respiratory function through exercises to strengthen the inspiratory and expiratory muscles. In this phase, various walking support braces were used, including a Peromed brace, Codivilla spring, and finally an Ottobock brace (Ottobock, Budrio, Italy), which the patient currently wears.

On August 14, the patient was discharged with the indication to continue rehabilitation. In September, he came to our center to begin motor reconditioning and restoration of activities of daily living (ADL). Upon initial examination, he suffered major functional impairments including early fatigue, loss of muscle mass and strength, balance impairment, and gait disturbances due to bilateral damage of IPSN and EPSN for which he required the continued use of orthoses and the Ottobock braces.

### Instrumental evaluation

The patient signed an informed content and underwent a battery test with regard to respiratory, metabolic, and neuromuscular domains. The evaluation of the respiratory function was based on a spirometry examination and included the vital capacity (VC), residual volume (RV), total lung capacity (TLC), Motley index (MI), forced expiratory volume in 1 s (FEV1), FEV1/VC, and diffusing capacity for carbon monoxide (DLCO). The metabolic evaluation was based on indirect calorimetry (QRNG, COSMED, Milano, Italy) and bioimpedance (Akern, Pontassieve, Italy) and included the resting metabolic rate (RMR), respiratory quotient (RQ), resistance (Res), reactance (Rea), phase angle (PA), body water (BW), fat mass (FM), free fat mass (FFM), body cellular mass (BCM), and extracellular mass (ECM). Lastly, the evaluation of the neuromuscular function was based on electromyography and included the nerve conduction velocity (NCV), latency (La), and amplitude (Amp). The pre- and post-treatment values are summarized in Table 1.

**TABLE 1:** Absolute and percentage changes of the functional parameters measured at the beginning and at the end of the AMA intervention.

Functional test and parameter	T0 evaluation	T1 evaluation	Modification (%)
Spirometry			
Vital capacity (L)	3.45	4.84	+40
Residual volume (L)	1.99	1.95	−2
Total	544	6.79	+25
Lung capacity (L)			
Motley index (%)	37	29	−21
FEV1 (L)	3.18	4.10	+28
FEV1/vital capacity (%)	92	85	−7
DLCO (mmol/min/kPa)	3.23	6.25	+93
Indirect calorimetry RMR			
(kcal/day)	1326	1816	+37
Respiratory quotient	0.92	0.8	−13
Bioimpedance			
Resistance (Ohm)	416	432	+4
Reactance (Ohm)	26	45	+73
Angle phase (°)	3.6	5.9	+63
BW and hydration (%)	60	46	−23
Fat mass (kg, %)	11.6–15	25.1–27	+116
Free fat mass (g, %)	63.9–85	66.4–73	+4
Body cellular mass (kg, %)	24.4–38	35.5–53	+46
Extra cellular mass (kg)	39.5	30.9	−21
Electromyography (IPSN dx/sx)			
Conduction velocity (m/s)	34.3/31.4	30.7/34.6	−10.5/+10.2
Latency (ms)	9.1/4.4	6.6/5.1	−27.5/+15.9
Amplitude (mV)	0.6/0.6	0.4/2.1	−33.3/+250

FEV1, forced expiratory volume in the 1st sec; DLCO, diffusing capacity for carbon monoxide; RMR, resting metabolic rate; BW, body water; IPSN, internal popliteal sciatic nerve.

### Adapted motor activity program

**FIGURE 1:**
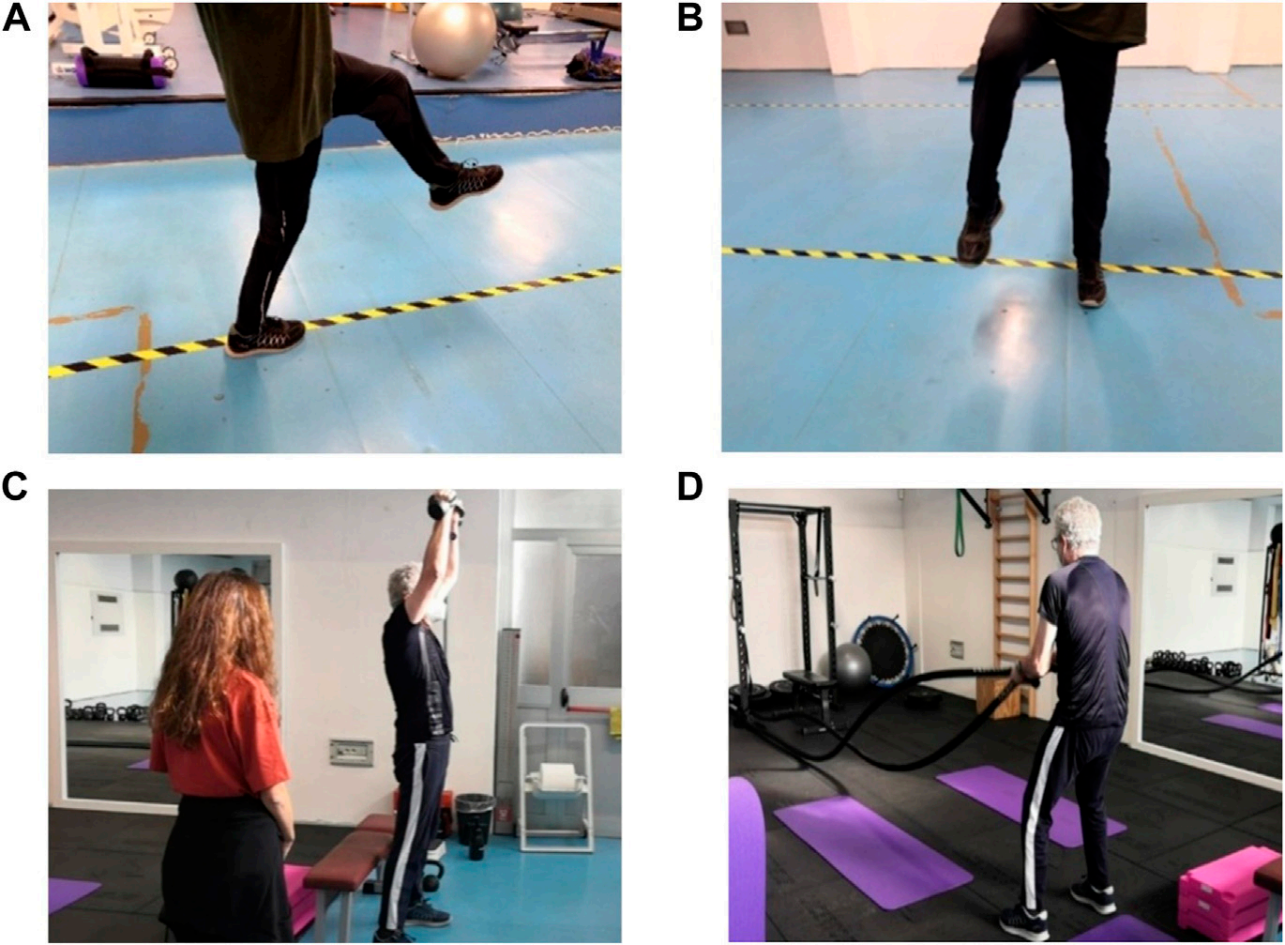
Execution of **(A)** frontal walk without sticks or braces; **(B)** side walk without sticks or braces; and **(C)** shoulder press with kettlebell. Next to the patient, an operator supervises the progress of the exercise; **(D)** alternating waves with a rope. All exercises refer to the third phase of the AMA program (with permission from the patient).

The AMA program required a combination of aerobic, strength, gait, and balance training. All sessions were carried out under the supervision of qualified operators (Figure 1). Based on the level of difficulty, modulated according to the patient’s observable functional improvement, the program can be divided in three phases.

First phase (September 2020–December 2020): Aerobic training consisted of 10 min of cyclette exercise at a power of 60 W. Strength training consisted of 20 min of exercises performed with low intensity and medium–high repetitions (three sets of 12–15 repetitions): bridge, total body resistance exercise (TRX) traction, step ups, and reinforcement of knee and ankle flexor and extensor muscles with elastic bands and inclined planks (three sets of 20 s). Gait training consisted of 10 min of gait alternating frontal walk, with complete roll of the foot, heel–toe walking, walking only on the toes with possible maintenance of the position at the stop, and side walking. Balance training consisted of 10 min of monopodalic work with support on the ground and bipodalic work on a trampoline, safely carried out with an espalier support. At the end of this phase, the patient gained amplitude in the articular range of motion (ROM), particularly for the ankle. Core strengthening exercises, such as bridge and plank, improved stability and helped make the patient’s gait tilt less. The use of sticks was progressively reduced until autonomous walking with braces.

**FIGURE 2:**
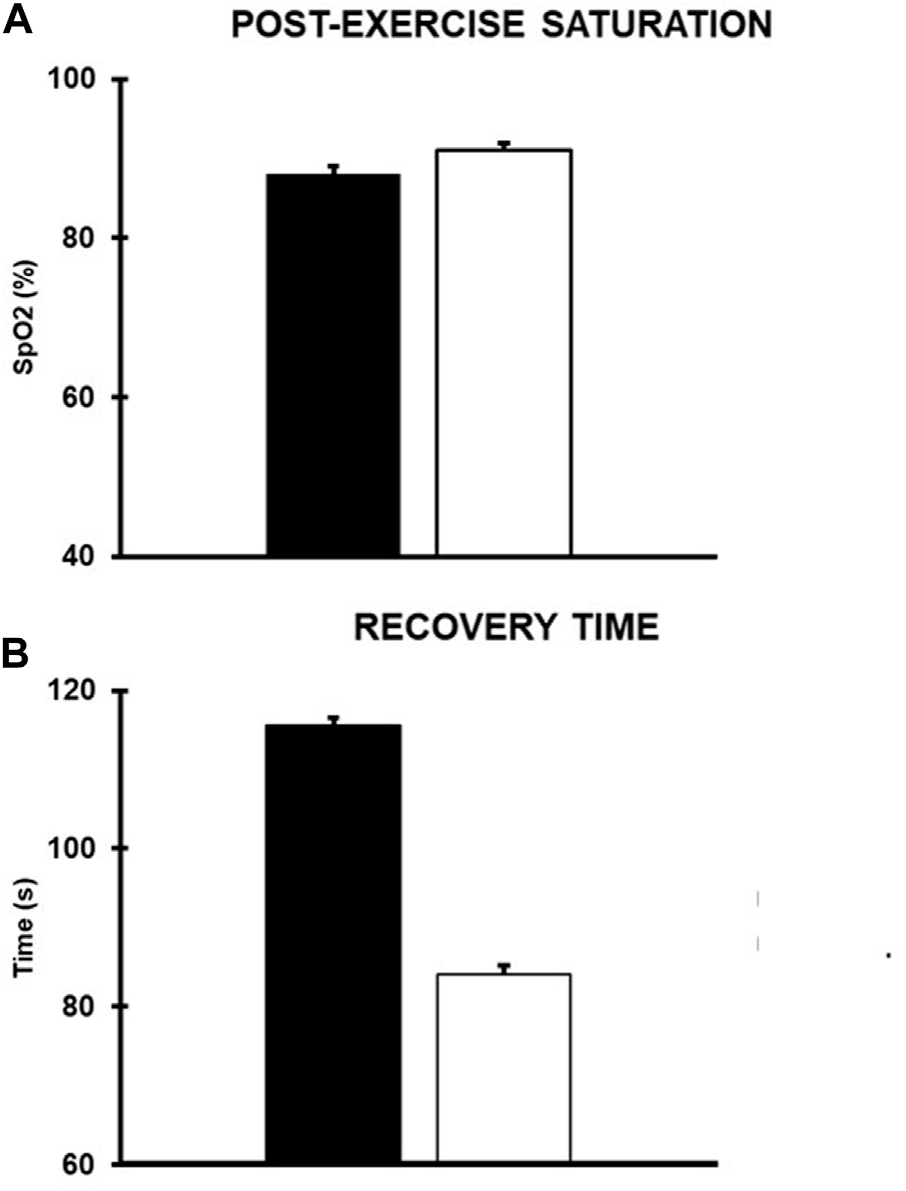
Mean values (SD) of the **(A)** post-exercise saturation and **(B)** the mean recovery time. Black and white columns refer to the mean values recorded in the first and last 2 months of the second phase of intervention, respectively.

Second phase (January 2021–April 2021): Aerobic training consisted of 10 min of rowing at a power of 80–100 W. Strength training consisted of 20 min of exercises with increasing loads and lower repetitions than the previous phase (three sets of 8–10 repetitions): double support squat with overload, bodyweight split squat, sidewalk with elastic band, and prone push up and plank (three sets of 30 s). Gait training consisted of 10 min of the same exercises of the previous phase, carried out with braces but without sticks. Balance training consisted of 10 min of monopodalic balance on a trampoline and bipodalic balance on unstable surfaces (Skimmy, Navaris) without espalier support. In this phase, for each workout, we measured the saturation level (SpO_2_) after the aerobic exercise and the relative recovery time, i.e., the time it takes to return to the SpO_2_ level recorded at rest (i.e., 99%). Regarding the mean values of the first 2 months of intervention and those of the last 2 months, it was appreciable that, although the post-exercise SpO_2_ values remained almost the same, a clear decrease in the recovery time occurred (Figure 2).

Third phase (May 2021–September 2021): Aerobic work consisted of 12 min of rowing at a power of 110 W. Strength training consisted of 20 min of exercises performed with increasing loads and lower repetitions than the previous phase (three sets of 5–6 repetitions): shoulder press with kettlebells, bench press with barbell or dumbbells, alternating waves with the rope, pull up with the support of elastic bands, double support squat with overload, TRX single leg squat, and plank (three sets of 40 s). Gait training consisted of 10 min of the same exercises of the previous phase carried out mostly without braces. Balance training consisted of 10 min of complex tasks such as maintaining balance on the trampoline while reaching for an object placed on the ground, and in monopodalic equilibrium, the patient had to touch objects positioned around him with his free limb. At the end of this phase, the patient showed increased autonomy in walking without braces, a noticeable improvement in strength, and good balance even in single stance.

## Discussion

### Results and considerations

COVID-19 has led to many infections and victims around the world (Pollard et al., 2020). Due to the severity of the disease, a high percentage of these patients require hospitalization in ICU, which is associated with a high risk of developing CIP and CIM (Tsai et al., 2004; Leung et al., 2005; Algahtani et al., 2016). Therefore, given the high number of potential patients, it appears essential to find rehabilitation strategies useful to counteract the long-term effects of both respiratory and neuromuscular diseases. Studies concerning the rehabilitation of CIP and CIM patients are focused on the treatment of the disease in the acute and immediate post-acute phases (Doherty & Steen, 2010; Nordon-Craft et al., 2011; Jang et al., 2019). Available data emphasize the importance of early diagnosis and mobilization and the need to support specific pulmonary rehabilitation in addition to physical therapy (Ydemann et al., 2012; Jang et al., 2019; Cheung et al., 2021). To the best of our knowledge, our study is the first to report the effects of an exercise-based program to contrast the long-term effects of CIP and CIM in a COVID-19 patient discharged from the ICU. After 1 year of training on a twice-weekly basis, we observed significant improvements in gait, balance, metabolism, body composition, and respiratory function but no ameliorations in neuromuscular function.

In particular, regarding the respiratory function, a large improvement in VC was seen (+40% on the initial value). The decrease in RV suggested an improvement in the capacity of air mobilization during maximal exhalation. TLC increased by 25% and the MI, a sign of pulmonary hyperdistension due to broncho-obstruction or pulmonary emphysema, decreased by 21%; FEV1/VC returned to the physiological range. The improvement of lung parameters led to a decrease in the recovery time of the patient after any effort (Figure 2). The DLCO value remained low despite the other volume’s improvement, likely due to the presence of fibrotic tissue in the upper and lower lung lobes as observed in a CT scan performed in April 2021. The general amelioration of spirometric parameters seems to confirm the role of aerobic exercise in improving the lung function in post-COVID-19 patients (Araùjo et al., 2022).

Data on metabolism and body composition underpinned a substantial improvement in all parameters. RMR increased by 37%, with a 4% increase in FFM. RQ decreased, approaching the value expected in a healthy subject at rest (Leff et al., 1987). PA, BW, and BCM returned within the physiological ranges. FM, which at T0 was critically low, increased by 116% at T1. Improved body composition and increased muscle mass appear to be in line with literature data, suggesting the role of physical activity as an effective means of treating sarcopenia (for review, see Montero-Fernández & Serra-Rexach, 2013).

Electromyographic parameters were unchanged. The EPSN was bilaterally absent in both evaluations. At T1, left IPSN had a slight improvement in NCV and Amp but a slight worsening in La, while right IPSN had a slight improvement in La but a slight decrease in NCV and Amp. This seems coherent with a follow-up study (sample of 22 subjects) reporting that denervation of muscle consistent with previous CIP can be found up to 5 years after ICU discharge in >90% of these subjects (Fletcher et al., 2003). To the best of our knowledge, the present study is the first to show that AMA does not improve neuropathy in this kind of patients.

The result of the proposed intervention is a clinical and functional improvement which reverberates in an important increase of autonomy during ADL. As mentioned, the use of sticks while walking was gradually reduced until abandonment, and to date, the patient shows good autonomy, albeit not total, in ambulation without braces, without dyspnea or early fatigue. Wintermann et al. (2018) highlighted the disabling impact of fatigue in this population of subjects, showing that patients with CIP and CIM report signs of chronic fatigue even 6 months after ICU discharge. This seems to be in line with the case reported, as at a first evaluation, the subject complained of a markedly premature fatigue. Possibly, its gradual decrease could be linked to the decreased recovery time after an effort (Figure 2). Furthermore, he restored the ability to autonomously carry out some fundamental activities of social life such as driving a car. The good outcomes of the training program could also rely on the constant subject supervision during the activities. This is in line with other studies (Lacroix et al., 2017; Minetama et al., 2019), showing a greater level of effectiveness of supervision compared to unsupervised physical exercise in different populations of subjects.

### Limitations

Our data, reported for a single case, cannot be generalized to other patients with CIP and CIM and do not allow us to accurately quantify how much the reported improvements derive from the proposed intervention or from a spontaneous recovery. However, previous follow-up studies focusing on the long-term consequences of CIP and CIM provide conflicting data or are in need of further investigation. Lacomis et al. (1998) have found similar functional outcomes in patients with CIM and CIP. Instead, the study by Guarneri et al. (2008) reports that the presence of CIM alone has generally a good prognosis, while CIP or the simultaneous presence of CIP and CIM is more likely to lead to long-term consequences and suggests that a possible explanation for these different results is that, in the aforementioned study, patients were followed up for only 4 months, when CIM is still prevalent. Finally, a recent scoping review by Intiso et al. (2022) concludes that subjects with intensive care unit-acquired weakness (ICUAW) may show improvements at the follow-up but that due to short follow-ups and the paucity of defined outcome measures, confirmations are required. Certainly, future studies on a large sample and with a control group will be needed to confirm or disprove the results obtained in this study.

## Conclusion

This work shows that supervised AMA can be an effective and safe tool to improve respiratory, metabolic, and functional conditions, unlike neuropathy, in this kind of patient.

## Data Availability

The raw data supporting the conclusion of this article will be made available by the authors, without undue reservation.

## Abbreviations

ADL, activities of daily living; AMA, adapted motor activity; BCM, body cellular mass; BW, body water; CIM, critical illness myopathy; CIP, critical illness polyneuropathy; CPAP, continuous positive airway pressure; ECM, extracellular mass; EPSN, external popliteal sciatic nerves; FFM, free fat mass; FM, fat mass; ICU, intensive care unit; MI, Motley index; NCV, nerve conduction velocity; PA, phase angle; PEG, percutaneous endoscopic gastrostomy; RMR, resting metabolic rate; ROM, range of motion; RQ, respiratory quotient; RV, residual volume; TLC, total lung capacity; VC, vital capacity.
